# Atomistic details of the phosphodiester cleavage of ribonuclease H

**DOI:** 10.1186/1758-2946-3-S1-P25

**Published:** 2011-04-19

**Authors:** B Elsässer, G Fels

**Affiliations:** 1 University of Paderborn, Department of Chemistry, Warburgerstr. 100, 33098 Paderborn, Germany

## 

RNase H belongs to the nucleotidyl-transferase (NT) superfamily and in the presence of divalent metal ions, preferably Mg^2+^ it catalyzes the hydrolysis of phosphodiester linkages of the RNA strand in the DNA:RNA hybrid duplex. RNase H activity is encoded as a part of the reverse transcriptase (RT) that converts a retroviral single strained RNA genome into double strained DNA. Due to the RNase H activity in HIV reverse transcriptase (HIV-RT), it represents a promising target for anti-HIV drug design.

In our study we focused on a computational investigation of the hydrolytic mechanism of *human* RNase HI (PDB Code 2QKK) [1] using a comprehensive QM/MM theoretical method that is based on DFT/B3LYP calculation of the interactions in the QM region and the inclusion of the interactions of the surrounding protein and solvent water in the MM region as implemented in the software package of NWChem [2].

Starting from the X-ray structure of the mutated enzyme-substrate complex we changed the enzyme into the active form to reach the reactant state. Furthermore, this structure has been validated by additional docking studies. Afterwards, using a series of constrained and relaxation steps we could model the reaction path, identify the product state and found a stabile intermediate state along the reaction coordinates (Fig. 1).

**Figure 1:**
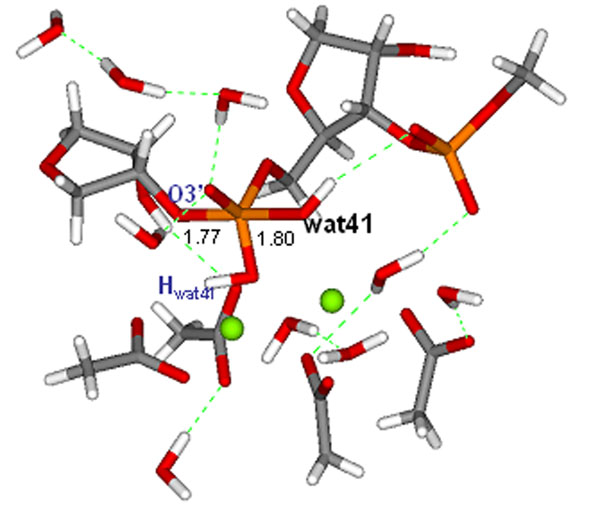
Stabile pentacoordinate intermediate over the RNase H pathway.

After a nucleophilic attack of a water molecule on the scissile phosphorous a water-proton is transferred to the O1P oxygen as the first step.

In the second, consecutive step the proton is shuttled to the O3’ oxygen and the nucleotide is being cleaved to form the product state. Finally, we performed transition state search and energy barrier calculations over the reaction coordinates and identified the rate limiting step of the reaction.The calculated reaction energy is in excellent agreement with experimental findings.

## References

[B1] Nowotny M Gaidamakov SA Ghirlando R Cerritelli SM Crouch RJ Yang W Structure of human RNase H1 complexed with an RNA/DNA hybird: insight into HIV Reverse Transcription Mol Cell 2007 28 264 276 10.1016/j.molcel.2007.08.015 17964265

[B2] Valiev M Bylaska EJ Govind N Kowalski K Straatsma TP van Dam HJJ Wang D Nieplocha J Apra E Windus TL de Jong WA NWChem, a comprehensive and scalable open-source solution for large scale molecular simulations Comput Phys Commun 2010 181 1477 10.1016/j.cpc.2010.04.018

